# Significance of penile hypersensitivity in premature ejaculation

**DOI:** 10.1038/s41598-017-09155-8

**Published:** 2017-09-05

**Authors:** Liqiang Guo, Yuqiang Liu, Xuesheng Wang, Mingzhen Yuan, Yang Yu, Xiulin Zhang, Shengtian Zhao

**Affiliations:** 1 Department of Urology, the Second Hospital of Shandong University, Jinan, Shandong 250033 P.R. China; 2 Institute of Urology, Shandong University, Jinan, Shandong 250033 P.R. China; 3 The Affiliated Hospital of Shandong University of Traditional Chinese Medicine, Jinan, Shandong 250014 P.R. China

## Abstract

To assess the correlation between penile hypersensitivity and premature ejaculation (PE), a total of 420 consecutive subjects attending our andrologic clinic for suspected PE were enrolled. The entire cohort was asked to complete the self-report intravaginal ejaculation latency time (IELT) by stopwatch. According to the IELT, the subjects were classified into 3 groups. Vibratory thresholds were recorded at the glans penis and penile shaft using a biothesiometer. We found that vibratory thresholds in the glans penis and penile shaft were significantly lower in both mild and severe PE group than in the control group (3.81 ± 0.57 and 3.54 ± 0.43 vs 4.73 ± 0.77 for glans penis p = 0.000; 3.64 ± 0.52 and 3.37 ± 0.50 vs 4.62 ± 0.69 for penile shaft p = 0.002). The vibratory threshold decreased as the disease aggravated. In the mild and severe PE groups, a significant positive correlation was detected between the mean values of IELT and the vibratory thresholds. Furthermore, in the receiver operating characteristics curve analysis, the area under the curve of the glans penis and penile shaft vibratory thresholds predicting severe PE were 0.852 and 0.893 respectively. Our study established a dose-dependent association between penile vibratory threshold and PE. Therefore, the vibratory threshold can serve as a potential marker for predicting the severity of PE.

## Introduction

Premature ejaculation (PE) is the most common sexual dysfunction with a prevalence ranging from 20% to 30% of the general male population with regards to subjective complaints^1, 2^. Despite its high prevalence, a universally accepted consensus of the definition of PE is lacking. Until 2008, the International Society of Sexual Medicine (ISSM) proposed a multivariate evidence-based definition of lifelong premature ejaculation: a male sexual dysfunction characterized by ejaculation which always or nearly always occurs prior or within about 1 min of vaginal penetration; an inability to delay ejaculation on all or nearly all vaginal penetrations, and negative personal consequences such as distress, bother, frustration, and/or the avoidance of sexual intimacy^3^. Acquired PE is characterized by gradual or sudden onset over the lifetime of the individual, who describes previous normal ejaculatory performance and ejaculates within approximately 3 min after vaginal penetration^4^. Based on the ISSM definition of PE, in terms of an IELT of about 1 minute, the prevalence of lifelong PE is about 4% of the general population^5^.

Classically, PE was thought to be psychologically or interpersonally based^6^. Recently, many other biological factors have been proposed to explain PE including genetic vulnerability^7, 8^, robust cortical representation of the pudendal nerve^9^, disturbances in central serotonergic neurotransmission^10, 11^, and thyroid diseases^12, 13^. In addition, it was considered that penile hypersensitivity may be one of the main pathologic mechanism of PE^14^. However, the link between penile hypersensitivity and other potential causes is controversial and lack a definitive conclusions. A case–control study conducted by Xin suggested that PE patients had hypersensitivity in the glans penis and penile shaft^14^. However, other investigations did not show a correlation between penile sensitivity and PE^15, 16^. Interestingly, with quantitative sensory testing analysis, Salonia *et al*. found that PE patients had penile hyposensitivity, instead of penile hypersensitivity^17^. One potential reason for the inconsistency is that most of the studies predated the new ISSM definition of PE and, as such, the definitions of ‘normal’ and PE were somewhat arbitrary and variable across studies. In fact, in many clinical trials and neurobiological evaluations of the population of men with PE have been largely self-diagnosed, and thus are somewhat heterogeneous and, poorly defined, making a comparison between studies difficult. In addition, the relatively small sample size contributed to the conflicts among studies. In the present study, we used a penile biothesiometer to reassess the correlation between penile hypersensitivity and lifelong PE in a relatively large cohort, as defined by the criteria identified by the ISSM definition of PE.

## Materials and Methods

### Study population

A consecutive 420 subjects attending our andrologic clinic were evaluated for suspected PE. After the initial sexological assessment, 83 did not fulfill the inclusion criteria and were excluded from the study protocol leaving a final 337 participants for enrollment. Two hundred and thirteen subjects were diagnosed as lifelong PE and were included in the lifelong PE group. Lifelong PE was diagnosed by an experienced andrologist on the basis of the International Society of Sexual Medicine (ISSM) definitions^3^, including the evaluation of intravaginal ejaculatory latency time (IELT) by stopwatch^18^. Pre-assessment IELT was measured for a 4-week baseline period during which all participants were asked to have sexual intercourse at least four times. According to the IELT, the lifelong PE participants were divided into two groups: the mild PE group included 152 participants with IELT ≥30 s and ≤60 s and the severe PE group included 61 participants with IELT < 30 s. The remaining 124 men did not fulfill the criteria either for a diagnosis of PE, or for a diagnosis of other sexual dysfunctions and were considered as controls. Both patients and controls underwent a comprehensive medical and sexual history and a detailed physical examination, including a complete neurological assessment. The participants were selected based on the following inclusion criteria. (1) were >18 years old, (2) were in a stable heterosexual relationship with a sexually active partner for at least 6 months preceding enrollment, (3) were healthy, without any neurological, metabolic, endocrine disorders, genital tract infection, or any organic disorder, (4) were not suffering from any Diagnostic and Statistical Manual of Mental Disorders-IV axis I disorder, (5) did not complain of any organic cause of PE, including anatomical abnormalities, (6) had a normal erectile function (IIEF-15 score > 26), and (7) did not have a history or were not currently abusing alcohol (drinking alcohol almost everyday) or using illicit drugs. This study received ethical committee approval from the Ethics Committee of the Second Hospital of Shandong University with written informed consent obtained from each participant prior to enrollment. The participants were aware that their participation in the study was voluntary. The methods were carried out in accordance with the approved guidelines.

### Methods

Penile skin vibratory sensitivity thresholds were assessed by means of a biothesiometer (Sensiometer A200, Laxons Technology Co., Ltd, Beijing, China), a tool that delivers vibratory stimuli and simultaneously quantitatively measures peripheral sensory thresholds at different genital sites. Each participant was placed in a supine position, in a quiet room with a consistent temperature of 25 °C with a black patch covering his eyes^19, 20^. Hand vibratory sensation thresholds were first tested at the pulp of the right index finger. Finger tip measurements are considered useful in providing information about general neuropathy. In addition, the patient was relaxed before the test on the penis. The measurements then began at the genital level, including the glans penis and penile shaft. The vibratory sensation probe was applied in contact with the penis with its own weight to avoid variations in pressure.

The vibratory stimuli increased from 0 V until the subject first gradually sensed tiny synchronous prickle stimulations. Subsequently, the stimulation slowly was decreased until the subject reported the disappearance of the vibratory sensation. The rate of vibratory amplitude change was 0.1 V/second. The test was repeated 3 times, and all the critical values were recorded and averaged to estimate the sensory threshold of the glans penis and penile shaft. If the differences between two consecutive measurements at the same position were greater than 10%, they were tested again after a 5 minute interval. If the repeated test disparity was still greater than 10%, the subjects were excluded from this study to avoid possible subjects bias. The vibration frequency was fixed at 100 Hz, with a variable amplitude range of 0 to 72 μm. All procedures were performed by Dr. Yu who was blind to the sexual status of the participant by the same biothesiometer.

### Statistical analysis

The SPSS statistical software for windows version 18.0 (SPSS Inc., Chicago, IL, USA) was used for statistical analyses. The Kolmogorov–Smirnov test was applied to test normality. All data were presented as means ± SD or percentage. Statistical significance was determined using multiple comparisons between the groups performed by a one-way ANOVA, or the Chi-square test (χ^2^). Spearman’s correlation analysis was used to detect the relationship between vibratory thresholds and IELTs. To estimate the sensitivity value as a severity marker of PE, receiver operating characteristic (ROC) analysis was performed. P < 0.05 was considered statistically significant.

## Results

**Figure 1:**
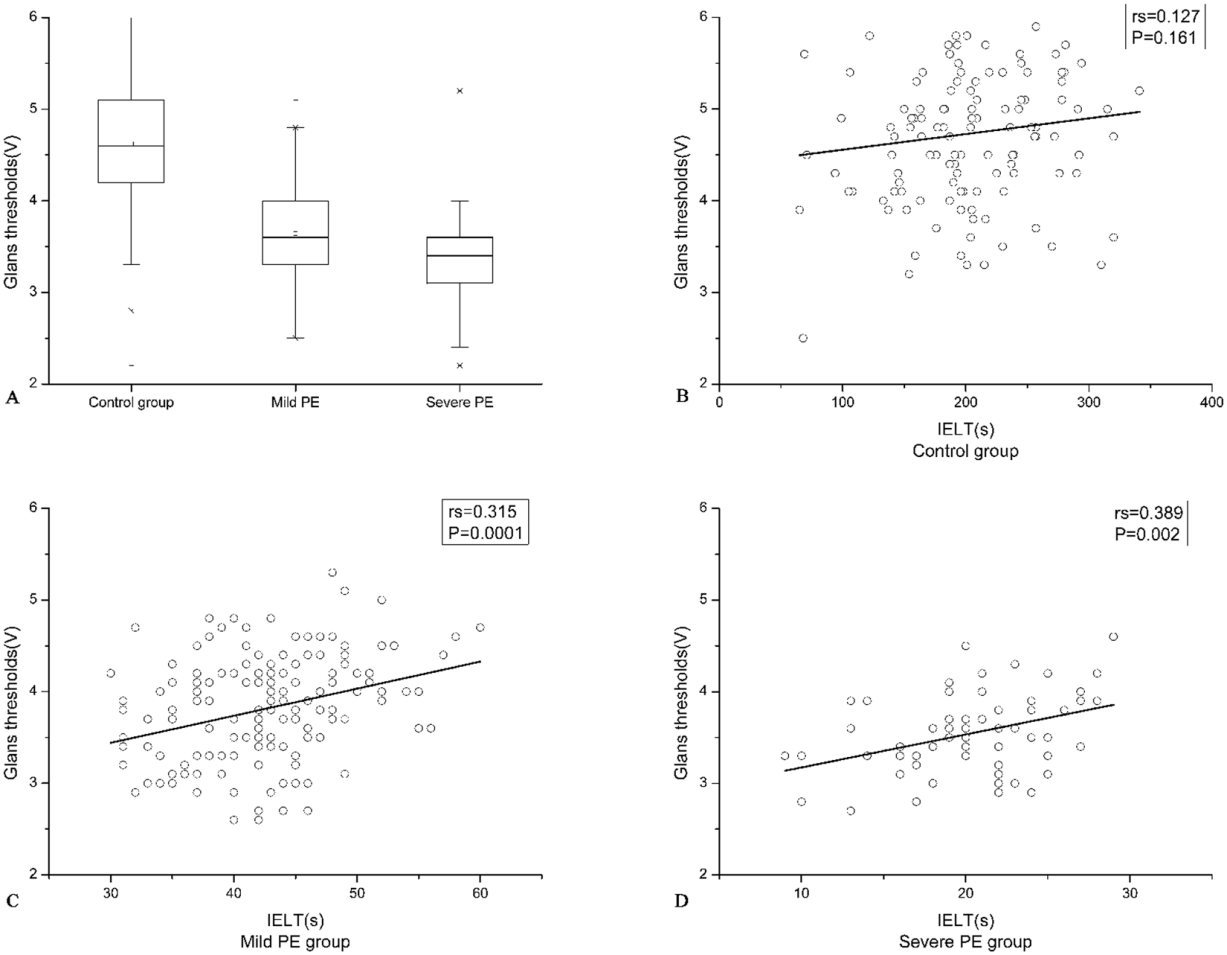
Difference in glans thresholds and Spearman’s correlation coefficient (rs) and the linear regression line between IELT scores and glans thresholds among the 3 groups.

**Figure 2:**
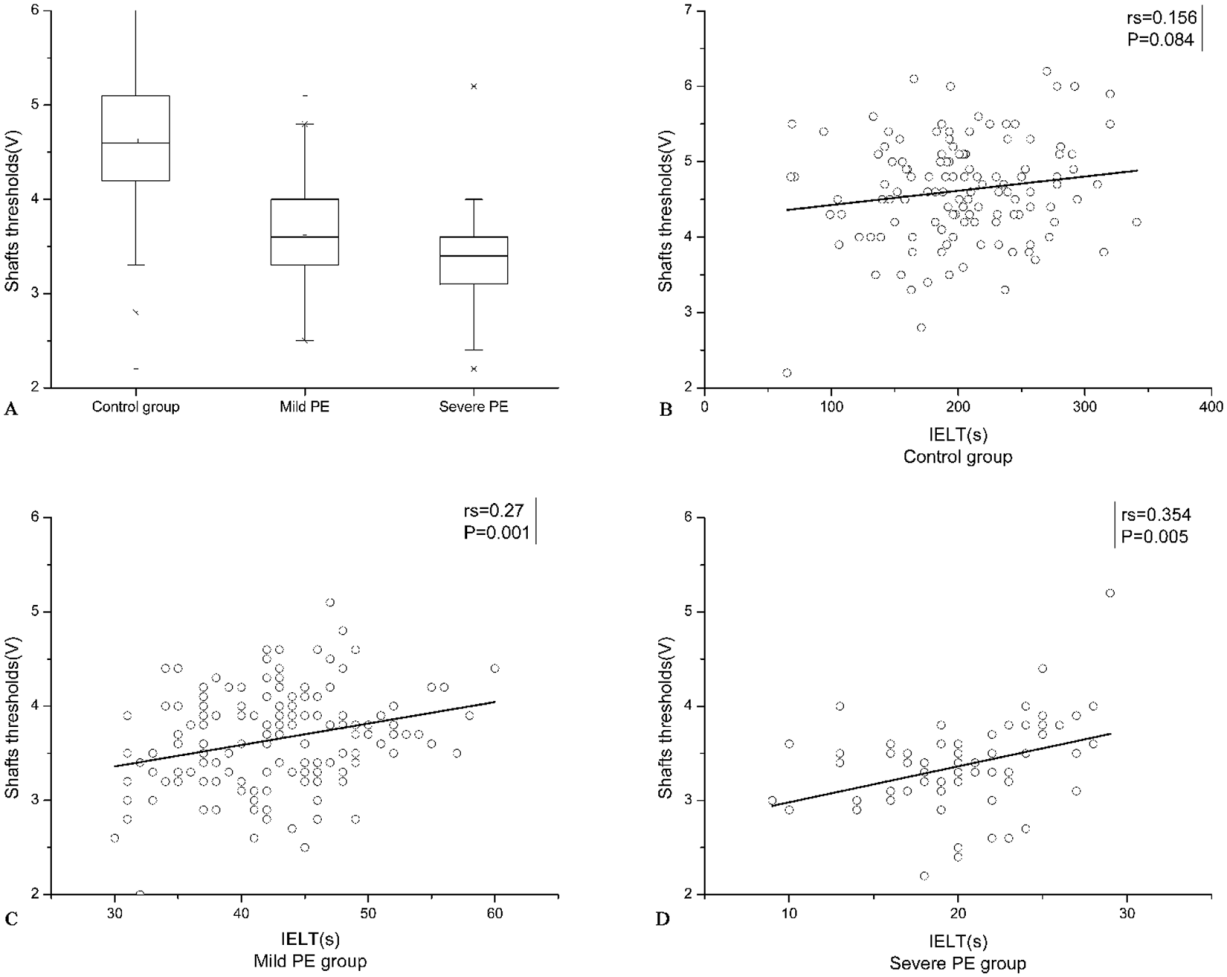
Difference in penile shaft thresholds and Spearman’s correlation coefficient (rs) and the linear regression line between IELT scores and penile shaft thresholds among the 3 groups.

The characteristics of the participants are shown in Table 1. Patients and controls were comparable with regard to age, BMI, tobacco smoking, alcohol abuse and IIEF-5 scores. The numerical variables were normally distributed (Kolmogorov-Smirnov test). Comparing the vibratory thresholds between the groups, all changes in the glans penis and penile shaft were statistically significant (Figs 1 and 2).

**Table 1:** Genaral characteristics of the patients and the control group.

	Control group (n = 124)	Premature ejaculation (n = 224)		P -value
		Mild PE (n = 152)	Severe PE (n = 61)	
Age (years)	32.56 ± 5.13	31.55 ± 5.26	29.03 ± 4.79	0.702
BMI (kg m^−2^)	24.09 ± 2.64	24.49 ± 3.11	23.76 ± 2.87	0.075
Smoking (%)	35/124(28.2%)	49/152(32.2%)	22/61(36.1%)	0.537
Drinking (%)	8/124(6.5%)	16/152(10.5%)	5/61(8.2%)	0.483
IELT (s)	202.12 ± 57.1	42.36 ± 6.11	20.24 ± 4.60	0.000*
Glans thresholds (V)	4.73 ± 0.77	3.81 ± 0.57	3.54 ± 0.43	0.000*
Penile shafts thresholds (V)	4.62 ± 0.69	3.64 ± 0.52	3.37 ± 0.50	0.002*
IIEF-5	23.47 ± 0.93	23.24 ± 0.90	23.36 ± 0.91	0.623

Values are given as mean ± SD. BMI: body mass index, IELT: intravaginal ejaculatory latency time, IIEF-5: International Index of Erectile Function-5. *Denotes statistically significant difference by one-way ANOVA.

To evaluate the possibility of the vibratory thresholds as a severity marker of PE, a correlation analysis using Spearman’s rank correlation coefficient was performed to assess the relationship between vibratory thresholds and IELT. The glans penis threshold levels were positively correlated with the IELT score for both the mild PE group (rs = 0.315; P = 0.0001) and the severe PE group (rs = 0.389; P = 0.002), without a significant correlation with the control group (rs = 0.127; P = 0.161) (Fig. 1). In addition, penile shaft threshold levels were positively correlated with IELT for the mild PE group (rs = 0.27; P = 0.001) and the severe PE group (rs = 0.354; P = 0.005), while there was no significant correlation with the control group (rs = 0.156, P = 0.084 (Fig. 2).

**Figure 3:**
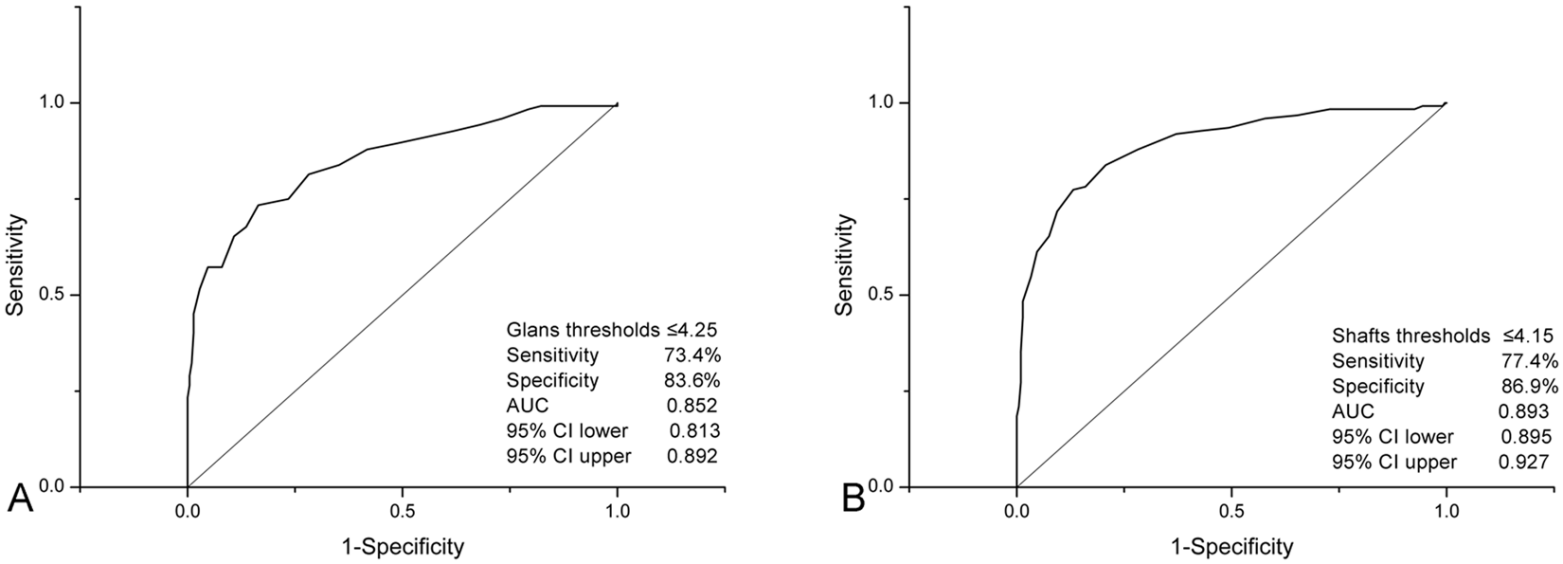
Receiver operating characteristic curve of glans and penile shaft thresholds for predicting premature ejaculation. AUC indicates area under the curve, CI: confidence interval.

In the ROC curve analysis shown in Fig. 3, the cut-off glans thresholds value for PE was 4.25 V (AUC: 0.852, 95% CI: 0.813–0.892). This value had a sensitivity of 73.4% and a specificity of 83.6% for differentiating the status of PE. The ROC curve analysis in the PE group identified a shaft threshold value of ≤4.15 V as a potential cut-off level for the prediction of the severity. The value had an AUC of 0.893 (95% CI: 0.895–0.927), with a sensitivity of 77.4%, and a specificity of 86.9% (Fig. 3).

## Discussion

Premature ejaculation is a sexual dysfunction that affects both a couple’s relationship and pleasure/enjoyment with a relative high prevalence^21^. Based on controlled clinical and epidemiological stopwatch studies, Waldinger and Schweitzer^22^ proposed a new classification of PE in which four PE subtypes were distinguished on the basis of IELT duration, frequency of complaints, and course in life, which included variable PE and subjective PE in addition to lifelong PE and acquired PE. Although men with lifelong and acquired PE appear to share the dimensions of short ejaculatory latency, reduced ejaculatory control, and the presence of negative personal consequences, Men with acquired PE are older, have higher incidences of erectile dysfunction, comorbid disease, and cardiovascular risk factors, and have a longer intravaginal ejaculation latency time (IELT) as compared with men with lifelong PE^23^. Acquired PE is commonly due to sexual performance anxiety^24^, erectile dysfunction (ED)^25^, and occasionally prostatitis^26^ or hyperthyroidism^12^. Because of the heterogeneous physiology of ejaculation and pathophysiology of different types of PE, the present study only evaluated the patients with lifelong PE. Our study showed that patients with lifelong PE had a penile hypersensitivity in terms of peripheral sensory thresholds, and we speculated that penile hypersensitivity may be a physiological trait that sets lifelong PE apart from other forms of PE.

In this study, we used a biothesiometer to assess the peripheral sensory thresholds for the modalities of vibration. Overall, our results suggested that PE patients showed a peripheral hypersensitivity, both at the glans penis and the penile shaft levels. Our results were in accordance with previous studies reported by Xin *et al*.^14^. In that study, vibratory thresholds of a series of 120 patients with PE and 66 healthy controls were evaluated using a biothesiometer, They found that the vibratory thresholds on the penile shaft and glans penis were decreased in patients with primary PE (with an mean IELT of 1.1 min, range 0–3), suggesting a role for vibratory thresholds in understanding the etiology of lifelong PE. However, this study may have an inherent operational bias as the vibrator was hand-held (allowing possible variation in the pressure applied) and the stimuli were presented in a non-random order^27^. On the other hand, Rowland *et al*.^16^ tested whether penile sensitivity to vibrotactile stimulation could distinguish premature ejaculators from normal men, by measuring penile vibratory thresholds in four small groups of men: primary PE (IELT 0–2 min), secondary PE and erectile dysfunction (ED,IELT 0.5–2 min),ED alone (IELT 5–7 min),and controls with normal sexual function (IELT 7–10 min). Although, their results argued against premature ejaculators having penile hypersensitivity, they found a significant correlation between ejaculation latency and threshold.

Furthermore, the study conducted by Salonia *et al*. showed that PE patients had significantly higher vibratory sensation thresholds for each site as compared with controls using a SMV-5 vibrometer^28^. This study evaluated 42 patients compared with 41 controls, demonstrating that the patients with lifelong PE might have a hypo- rather than hypersensitivity profile in terms of peripheral sensory thresholds. The authors acknowledged that their studies had several limitations and their results were difficult to interpret. The contradictory conclusion is made even more complex by the fact that the definition of PE has changed several times over the past few years. With different classification criteria, different studies may include a heterogeneous population of PE patients^14–17^, such as acquired premature ejaculation was not excluded in some studies^16^. Another important factor for the above inconsistent results may lie in the varied psychophysical methodology used for determining thresholds.

The present study found a correlation between self-reported IELT and penile sensory thresholds, with low thresholds related to short latencies in the PE group rather than in the control group. Furthermore, it was observed that the vibratory thresholds gradually decreased as the PE was aggravated both at the glans penis and the penile shaft. Therefore, the vibratory threshold can play a role as a severity prediction marker for the severity of lifelong PE. In the present study, the glans threshold cut-off value was 4.25 V with a sensitivity of 73.4% and a specificity of 83.6%. These results suggest that those men with a glans threshold of less than 4.25 V have an increased risk of PE. In fact, the combined application of glans thresholds and shafts thresholds could predict the severity of PE more efficiently and specificity. Based on the results of our study, we suggest that the risk of PE is higher when the glans threshold is ≤4.25 V and shafts threshold is ≤4.15 V in patients.

In this study, a biothesiometer was used to assess the peripheral sensory thresholds for the modalities of vibration. The biothesiometer is often criticized as unreliable or even dismissed because of several intrinsic limitations, such as amperage input and displacement output may be non-linear, stimulus presentation of ascending and descending magnitudes is considered problematic^19^, and it is subject to both experimenter and subject bias. However, in many studies in which biothesiometer were used as descriptor of sensory condition, repeatable results have been reported^14, 29, 30^. In our study, in order to obtain a more accurate threshold and minimize the subjective bias, the following procedures were strictly followed: (1) a black patch covered the eyes to avoid subjective influence from the participants, (2) the vibratory sensation probe was applied to contact with the penis with its own weight rather than hand pressure to avoid variations in pressure, (3) the vibratory stimuli were provided in an ascending manner until the first report of the sensation of vibration and then in a descending pattern from perception to no perception of vibration, (4) tests were repeated after a 5 minute break if the differences between two consecutive measurement for the same position were greater than 10%. If the repeated test disparity was still greater than 10%, the subjects were excluded from this study, (5) all procedures were performed by the same experimenter (Dr. Yu) who was blind to the sexual status of the participant, and (6) all participants were examined by the same biothesiometer.

In addition, the present study had several limitations. One limitation was the lack of serial detection of the vibratory thresholds in normal potent volunteers without ejaculatory disorders instead of the suspected PE control. Another limitation is that we did not design the “forced choice method” to establish a threshold. The “forced choice method” is generally considered an unbiased and reliable method for establishing threshold levels^31^. Even though it was not used in our study, since the six matters mentioned above were strictly followed during our evaluation, we believe the measured threshold was relatively reliable and had limited subjective impacts from the experimenter and participants. Nevertheless, a series of comprehensive blinded validation studies are warranted to confirm the use of vibratory thresholds in the clinical application in lifelong PE patients.

## Conclusions

Our study showed that patients with lifelong PE might have a hypersensitivity profile in terms of peripheral sensory thresholds. Penile hypersensitivity appeared to be a factor contributing to short intravaginal ejaculation latencies in the sample of lifelong PE men. Finally, the penile vibratory threshold is a valid measurement tool for predicting the severity of PE.
